# Identifying Gait-Related Functional Outcomes in Post-Knee Surgery Patients Using Machine Learning: A Systematic Review

**DOI:** 10.3390/ijerph20010448

**Published:** 2022-12-27

**Authors:** Christos Kokkotis, Georgios Chalatsis, Serafeim Moustakidis, Athanasios Siouras, Vasileios Mitrousias, Dimitrios Tsaopoulos, Dimitrios Patikas, Nikolaos Aggelousis, Michael Hantes, Giannis Giakas, Dimitrios Katsavelis, Themistoklis Tsatalas

**Affiliations:** 1 Department of Physical Education and Sport Science, Democritus University of Thrace, 69100 Komotini, Greece; 2 Department of Orthopedic Surgery, Faculty of Medicine, University of Thessaly, 41500 Larissa, Greece; 3 AIDEAS OÜ, 10117 Tallinn, Estonia; 4 Department of Computer Science and Biomedical Informatics, School of Science, University of Thessaly, 35131 Lamia, Greece; 5 Institute for Bio-Economy and Agri-Technology, Center for Research and Technology Hellas, 38333 Volos, Greece; 6 School of Physical Education and Sports Science at Serres, Aristotle University of Thessaloniki, 62110 Serres, Greece; 7 Department of Physical Education and Sport Science, University of Thessaly, 42100 Trikala, Greece; 8 Department of Exercise Science and Pre-Health Profession, Creighton University, Omaha, NE 68178, USA

**Keywords:** artificial intelligence, knee surgery, post-operative, walking, biomechanical data

## Abstract

Modern lifestyles require new tools for determining a person’s ability to return to daily activities after knee surgery. These quantitative instruments must feature high discrimination, be non-invasive, and be inexpensive. Machine learning is a revolutionary approach that has the potential to satisfy the aforementioned requirements and bridge the knowledge gap. The scope of this study is to summarize the results of a systematic literature review on the identification of gait-related changes and the determination of the functional recovery status of patients after knee surgery using advanced machine learning algorithms. The current systematic review was conducted using multiple databases in accordance with the PRISMA guidelines, including Scopus, PubMed, and Semantic Scholar. Six out of the 405 articles met our inclusion criteria and were directly related to the quantification of the recovery status using machine learning and gait data. The results were interpreted using appropriate metrics. The results demonstrated a recent increase in the use of sophisticated machine learning techniques that can provide robust decision-making support during personalized post-treatment interventions for knee-surgery patients.

## 1. Introduction

The human knee is the largest and most complex joint in the body. Its two articulations allow for greater flexibility but also make it more vulnerable to injuries. The knee is the most frequently injured joint in the human body, especially among young sports participants [1,2]. Most knee injuries can be treated conservatively, but surgical intervention may be necessary in some cases. These knee injuries can involve the ligaments, cartilage, tendons, or bones. In the United States, many studies on knee injuries among athletes have been conducted in collegiate settings. For example, an epidemiological study of volleyball male and female athletes in the National Collegiate Athletic Association found that the knee was the most commonly injured body part (16.3% in females; 25.5% in males), with different injury patterns for each sex [3]. In addition, knee joint degeneration and osteoarthritis (OA) are the most prevalent forms of disability, affecting millions of individuals worldwide [4]. When conservative treatment fails, preservation procedures such as knee osteotomies or joint replacement procedures (such as unicondylar and total knee arthroplasty, TKA) are the treatment of choice. TKA is the most common surgical procedure for people with end-stage OA, providing substantial improvement in quality of life [5].

Previous studies have shown that not all patients have the same rate of satisfaction after surgery and rehabilitation [6,7,8]. Approximately 11–20% of patients experience discomfort following TKA, which is related to persisting functional impairments [6]. The results do not seem to be enhanced by the adoption of more recent implant designs [7]. Alongside, the results after anterior cruciate ligament reconstruction (ACLR) can be poor, with an increased risk of ACL re-rupture and earlier onset of OA compared with healthy individuals [8]. These outcomes may be due to poor surgical outcomes, rehabilitation inadequacy, or treatment failure to restore healthy lower limb biomechanics, resulting in altered joint movement patterns, such as reduction in peak knee flexion angles during walking one year post-operation [9].

To aid clinical assessment, the identification of the post-operative rehabilitation status is important. Until now, performance and patient-based measures have been the usual methodologies regarding the evaluation of the recovery progress following knee surgery. Patient-reported outcome measures (PROM) are usually telephone interviews, mail-in questionnaires, or tools to analyze the health-related status of individuals that undergo knee surgery. Questionnaires, such as the Knee Injury and Osteoarthritis Outcome Score (KOOS) [10], International Knee Documentation Committee (IKDC) [11], Tegner-Lysholm knee scoring scale [12], and Tegner Activity Scale [13], have been extensively used in the literature. However, these measures rely on subjective statements presented by the patients or the primary caregivers and can be biased due to the recall of events or the raters’ subjective comments on patient performance. On the other hand, performance-based measures have been used for assessing mobility, range of motion, timing, and total elapsed time. Popular tests include the six-minute walk test (6MWT), stair climb test (SCT), chair stand test (CST), and timed up and go test (TUG) [14]. However, these tests are rated manually by another individual and may suffer from issues of subjectiveness and rater biases. Quantitative movement analysis has been suggested as an objective follow-up of biomechanical improvement and an additional factor for a more precise rehabilitation [15]. Conventional lab-based methods are time-consuming and require expensive equipment and specialized personnel. 

Thus, early and accurate evaluation of patients’ functional outcomes post-surgery would be essential for directing resources toward an effective and objective assessment. Machine learning (ML) is a data analysis approach that produces algorithms to predict outcomes and repeatedly “train” them from new data. It is increasingly emphasized in orthopedics [16] as a competitive alternative to regression analysis, capturing nonlinearities and complex interactions among multiple predictor variables [17]. Utilizing machine learning techniques to analyze specific kinematic and kinetic parameters permits a quick evaluation of the rehabilitation stage [18]. Additionally, the growing use of lightweight, inexpensive, and mobile wearable sensors (specifically inertial measurement units, IMUs) to extract meaningful movement characteristics can provide further useful information for clinical evaluation [19]. Deep learning algorithms have been used on IMU-based movement data to categorize and quantify activity-specific motions [20]. 

The aim of this review is to identify studies that have utilized machine learning (ML) or deep learning techniques to evaluate the rehabilitation stage of the knee joint following major orthopedic surgery using biomechanical data from gait analysis. Gait analysis was chosen as the focus of this review because it is a common task in daily activity, is easily performed, and is closely linked to clinical assessment of knee injuries. ML and deep learning techniques have the potential to significantly improve the accuracy and efficiency of gait analysis, making them valuable tools for monitoring and assessing the rehabilitation process following knee surgery. By identifying and reviewing relevant studies, this review aims to provide a comprehensive overview of the current state of the field and highlight the potential benefits and limitations of using these techniques in the evaluation of knee surgery rehabilitation. 

### Machine Learning in a Nutshell 

This section summarizes the important terminology with respect to ML models that were used in the research articles that were part of the present review. The purpose of this is to assist the readers’ knowledge and to ensure that everything is covered. ML is a subfield of artificial intelligence (AI) that deals with the creation of algorithms that can autonomously learn to generate accurate predictions by relying on their past experiences as opposed to pre-programmed instructions (Table 1).

## 2. Materials and Methods

### 2.1. Reporting

The criteria for recommended documentation items for systematic reviews and meta-analyses served as the basis for the conduct of this systematic review, which was carried out in accordance with PRISMA guidelines [21] (the current systematic review was not registered). Two authors (C.K., A.S.) independently completed the review procedure (literature search, selection of papers, and extraction of data). A third author addressed discrepancies (S.M.). 

### 2.2. Literature Search

The following databases were searched systematically: (a) MEDLINE (through PubMed), (b) Scopus, and (c) Semantic Scholar. In addition, a manual search was also conducted on Google Scholar to identify articles cited by the collected papers quoting the retrieved papers. Abstracts not published in English, conference abstracts, and the OpenGrey database were evaluated as grey literature. Initial determinations of the papers’ eligibility were based on their titles and abstracts. Then, the entire texts of the first qualifiers were examined to determine if they met the inclusion criteria. Table 2 lists the structured search technique for each database.

### 2.3. Eligibility Criteria

In this study, the population consisted of patients who had undergone knee surgery. The intervention was the surgery itself. There were no comparators, as the focus of the study was on the effects of the surgery. The outcomes of interest were whether the patient was healthy, had undergone total knee arthroplasty (TKA), or had an anterior cruciate ligament (ACL) injury. The study setting criteria were designed to diagnose the patients and assess the effectiveness of the surgery in improving their overall health and mobility.

The following criteria were employed for inclusion:

1. Articles which are employing ML techniques;
2. Studies that refer to knee injuries (e.g., anterior cruciate ligament, TKA, total knee replacement, meniscectomy, meniscal suturing, unicompartment knee arthroplasty, anterolateral ligament reconstruction, anterolateral ligament repair, posterolateral corner reconstruction, posterior cruciate ligament and high tibia osteotomy);
3. Studies that are based on gait-related biomechanical data.

The following criteria were used to exclude contributions: (i) articles published prior to 2015; (ii) studies without human subjects, studies which employed only statistical analysis; (iii) studies that examine movements unrelated to gait (e.g., countermovement jump); (iv) studies with only pre-surgery measurements with aim to predict the post-surgery recovery status; (v) studies without biomechanical data; (vi) articles not written in English; and (vii) editorials, commentaries, and book chapters.

### 2.4. Data Extraction

A modified Microsoft Excel document was used for the retrieved data. The category, author name, publication year, specification of the task, type of data, subjects, outcome assessment, feature engineering, machine learning algorithms, validation method, and results were provided for each of the retrieved paper. The list of the authors was also included.

### 2.5. Statistical Analysis

In this review study, accuracy was the most assessed evaluative metric in determining the predictive capabilities of the proposed learning algorithms.

### 2.6. Quality Assessment

The quality of non-randomized studies was evaluated using a modified methodologic index (MINORS) [22,23]. The following information was considered on a six-item checklist: performance metrics, dataset distribution, ground truth label determination, the feature set that was used as inputs, disclosure, and the aim of the study. Data were extracted and recorded using standardized forms.

The two observers worked to address differences in article selection, quality assessment, and data extraction (A.S., C.K.) and organized a meeting of consensus. The items were assigned a score of 0 (not reported), 1 (reported but inadequate), or 2 (reported but adequate). The mean modified MINORS score was 9.33 ± 1.75 across all investigations. It is important to note that the score range per item was in the range of (0,12).

**Figure 1:**
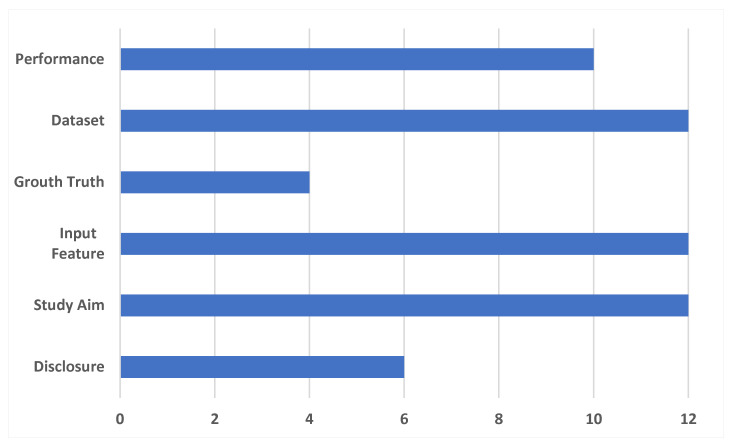
Quality assessment outcomes based on the MINORS tool.

As depicted in Figure 1, each of the six published studies explicitly described the study’s objective, input characteristics, and the dataset obtained using suitable metrics. In five investigations (83.33%), a clear description and distribution of the performance were given. One study (16.66%) precisely stated how the ground truth (AI’s reference standards) was established, whereas two utilized AI models that were improperly trained. Insufficient descriptions of ground truth assignments were the leading cause of quality point reductions. Finally, 50% of the employed studies failed to disclose a declaration of conflict of interest.

## 3. Results

**Figure 2:**
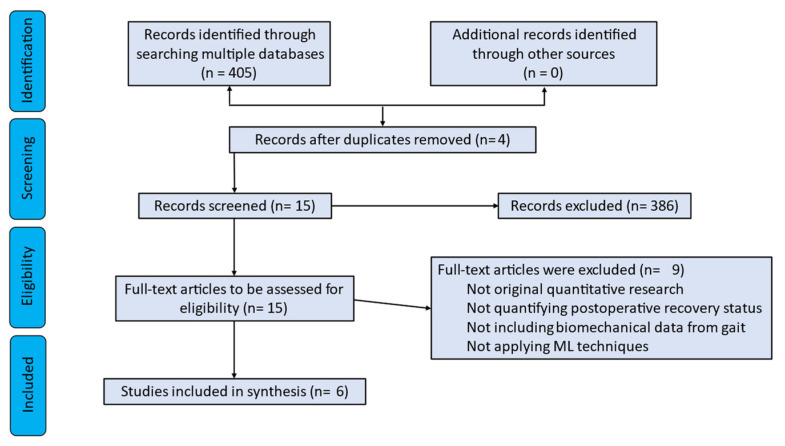
Flow chart of the proposed literature search.

In total, 405 articles were retrieved: 390 from Scopus, 14 from PubMed, and 1 from Semantic Scholar. The aforementioned inclusion/exclusion criteria led to 15 studies. Nine further papers were removed due to unrelated information (e.g., those concentrating on various movements or not quantifying post-operative recovery status). Taking into account all, six original articles were employed in this systematic review. The workflow diagram of the proposed literature search is depicted in Figure 2.

The included studies in this systematic review were classified into the following application domains: (i) TKA surgery (5 studies) and (ii) ACL surgery (1 study). For each study, the main results were quoted. The validity of each study is based on methodological weaknesses and strengths. In addition, significant methodological aspects of the aforementioned articles are discussed.

The employed studies in the current systematic review are listed in Table 3. In particular, it describes the recent use of ML models to the evaluation of the recovery status following knee surgery utilizing gait data as the primary data source. 

### 3.1. TKA Surgery 

In 2015, Kuntze et al., worked on the identification of multi-muscle activation patterns changes in 10 post-surgery female TKA patients during walking [28]. To achieve this goal, they recorded surface electromyograms (EMG) from 7 lower limb muscles. They employed a wavelet transform algorithm for feature engineering and an SVM classifier for machine learning. They demonstrated that the recognition rates for the VM and BF activation patterns were 68.4% and 73.7%, respectively. Hence, stated that altered muscle activations for the vastus medialis and biceps femoris, along with distinct between-group differences, were effectively noted. In another approach, Martins et al., proposed a gait analysis approach to determine differences and similarities in gait performance between three assistive devices in the rehabilitation process of TKA patients [27]. In order to attain this objective, they used postural control parameters, spatiotemporal, and symmetrical indexes in combination with feature reduction techniques such as nonlinear kernel-PCA (KPCA) and linear PCA. They used a multiclass SVM for classification, and this combination of KPCA and MSVM achieved an accuracy of 98%. Furthermore, Jones et al. proposed a machine learning approach in order to compare healthy, unicompartmental knee arthroplasty (UKA) and TKA patients (total subjects: 145) [26]. In their experimental study, the subjects walked on a treadmill, and 27 gait-related variables were employed for the classification task using decision trees classifiers. They stated that 5% of the healthy subjects were classified as UKA and 92% of them as UKA. Furthermore, DTs demonstrated that factors associated with the initial heel strike were frequently used to differentiate between TKA and UKA individuals. In addition, UKA subjects had a higher first peak force and a faster weight acceptance rate than the TKA subjects and were similar to healthy subjects.

Emmerzaal et al. investigated if the functional recovery status after TKA can be estimated by a classification model [24]. In total, 17 inertial measurement units were used to record different tasks (e.g., walking and descending stairs). A comparison investigation led them to the conclusion that an LR classifier trained on six weeks of post-operative biomechanical data during walking was responsive to changes at 3, 6, and 12 months post-TKA (with a 67.3% accuracy). In contrast with the other studies, Young-Shand et al. identified clusters among TKA candidates and postoperatively using knee biomechanical parameters during gait and demographic data and machine learning cluster analysis [25]. They performed 3D gait analysis on 134 pre- and 105 post-TKA patients, and they used PCA as a feature reduction technique. Using hierarchical agglomerative cluster analysis employing Ward’s minimum variance criteria, they identified four sex-specific and biomechanical clusters. It was finally concluded that patients’ cluster classification could be used for the formulation of personalized treatment strategies. They demonstrated that improvements in gait biomechanics following TKA were cluster-specific. Clusters 2 and 3 had the largest improvement in knee joint kinematics and kinetics after TKA, as compared with clusters 1 and 4, with superior function. In addition, none of the gait kinematic or kinetic factors improved from pre-TKA to post-TKA despite the fact that cluster 1 was small and statistical power was low.

### 3.2. ACL Surgery

In this category, only one study has been included. Specifically, Kokkotis et al. proposed an explainable ML approach to: (i) determine significant gait kinetic and kinematic parameters and quantify their relevance to the diagnosis of ACL injury and (ii) explore the variations in sagittal plane kinetics and kinematics data of the gait cycle between ACL deficient (ACLD), ACL reconstructed (ACLR) and healthy (control) subjects [29]. The study enrolled 151 subjects (ACLD 44, ACLR 54, and Control 53) with a total of 657 gait trials. The Relief algorithm was employed to decrease the feature dimensionality of the problem. Furthermore, a comparative analysis was finally performed to identify the best-performing classification model. For this aim, eight well-known classifiers were employed. The best score was achieved (94.95% accuracy) by the SVM classifier, which employed 21 biomechanical parameters. Furthermore, they discussed the impact of the identified parameters on the classification task via SHAP. Previous studies, which are based on traditional statistics, have noticed altered gait biomechanical parameters in ACL injuries [30,31,32]. On the 3-class problem, they demonstrated that the model output was significantly affected by the minimum knee flexion angle during the stance phase, maximum hip flexion angle during the swing phase, maximum plantar-flexion angle over the entire gait cycle, anterior (propulsive) GRF peak, second medial GRF peak, peak knee flexion angle during stance phase, maximum dorsiflexion moment during stance phase and first medial GRF peak biomechanical parameters. Most of these variables are consistent with the existing literature [30].

### 3.3. Subject Characteristics

In the TKA category, Emmerzall et al., used 20 healthy subjects as the control group, 19 subjects with unilateral knee OA and 17 of them, which were re-evaluated at 1.5, 3, 6, and 12 months post-TKA surgery with the main aim to develop an ML model for monitoring functional recovery status post-TKA [24]. The main inclusion criteria were knee OA and the wait time for total knee replacement. Young-Shand et al. employed 135 subjects 1 week before and 109 subjects one year post-TKA with the main aim to determine clusters among TKA candidates and compare gait differences between clusters post-surgery [25]. The main inclusion criterion was the ability to walk 6 m without a walking aid. Jones et al. performed gait comparisons of unicompartmental and TKA with healthy individuals. During their study, 145 subjects were enrolled, of which 121 were healthy, 12 with mobile-bearing medial UKA, and 12 with TKA. The inclusion criterion was a 12-month period after knee surgery. Martins et al. employed 13 elderly patients with KOA who were subjected to TKA in order to recognize similarities and differences in gait performance between 3 assistive devices [27]. Furthermore, Kuntze et al., worked with 10 post-TKA subjects and 9 control subjects in order to identify multi-muscle activation pattern alterations in post-operative female TKA patients [28]. They included subjects with the ability to accomplish activities of daily living. 

In contrast with the TKA studies, in the ACL category, Kokkotis et al. employed 151 subjects (ACLD: 44, ACLR: 54, and control: 53) in order to recognize significant biomechanical parameters associated with ACL injury [29]. The ACLD subjects participated in this study on average 30 days post-knee injury. The ACLR subjects participated for at least 6 months after the knee surgery, and the control subjects declared that they had no history of neurologic disorder or ACL of the lower extremities in the last 12 months.

## 4. Discussion and Conclusions

The current systematic review identified six original articles which showcased the current usage of AI tools in the challenge to quantify the recovery status of the rehabilitation process in post-knee surgery subjects. From the literature review, it emerged that this area of research is untapped. There is a gap in the existence of literature before 2019, which is possibly resulting from the limited computing power and the non-existence of big data in this field.

Patients’ enrolment: The enrolled populations in the identified studies varied in size, ranging from small samples of 13 patients to larger samples of 244 subjects. Most of the studies included patients with knee osteoarthritis (OA) before and after knee surgery, and some also included control subjects. One study included a subset of the sample that was re-evaluated at multiple time points post-total knee arthroplasty (TKA) surgery. Additionally, one study focused on comparing the gait characteristics of patients with mobile-bearing medial unicompartmental knee arthroplasty (UKA) to those of patients with TKA. Overall, the enrolled populations in these studies represented a diverse range of individuals with different diagnoses, conditions, and stages of recovery, providing valuable insights into the effects of different knee surgery interventions on gait and mobility.

**Figure 3:**
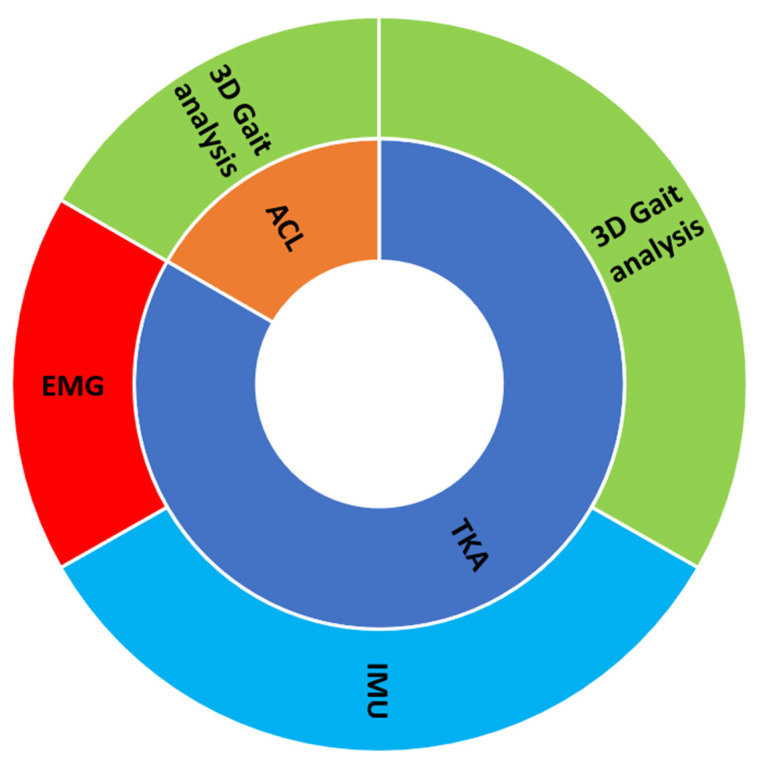
Type of data of the employed studies.

Input data: As input data, the majority of the studies used biomechanical data, which comes from a motion capture system (the Golden Standard System), and the capture type is called 3D clinical gait analysis [33]. Specifically, two studies in the TKA category and the only study of its kind in the ACL category employed this type of data. In contrast to the aforementioned studies, two of five studies in the TKA category used IMU data, and only one out of five studies used EMG from gait. (Figure 3). Specifically, the input data used in the studies include kinematic data (from IMU and camera-based systems), kinetic data, and EMG data during the gait cycle. These data were used to study various aspects of gait, such as gait biomechanics, gait patterns, muscle activations, and the effects of different assisted devices, among total knee arthroplasty (TKA) subjects, unicompartmental knee arthroplasty (UKA) subjects, subjects with acute anterior cruciate ligament deficiency (ACLD), subjects with anterior cruciate ligament reconstruction (ACLR), and control subjects.

*Learning, validation, and explainability*: Several ML models were used in the aforementioned studies. Three out of six studies applied SVM classifiers. One of them used a Logistic regression classifier, and another one used DT. As supervised learning, one out of six studies worked with hierarchical agglomerative (bottom-up) cluster analysis. Kokkotis et al. presented a comparative analysis. They used various ML models such as XGBoost, Random Forest, DTs, Naïve Bayes, SVM, KNN, LR, and NN [29]. 

Various validation strategies were recorded in the employed studies. The most frequent was the k-fold cross-validation strategy. Furthermore, one [29] of the six studies used a 70% training/30% testing validation strategy, and one of the six studies employed LOOCV.

Only one of the six studies utilized explainability methods, which play a crucial role in determining the reasoning behind the trained models’ decisions. Specifically, Kokkotis et al. [29] developed a comprehensive method for quantifying the input biomechanical factors’ contribution. They worked on a three-class problem to analyze the overall contribution of features, as well as on three (one-versus-one) binary SVM models emphasizing the contribution of features on each class. 

It is noteworthy that none of the employed studies were validated against an external dataset. Various limitations of the studies were highlighted. Various limitations were observed in the reviewed studies. The small number of employed subjects, the feature extraction [24], the non-use of transfer learning, the no existence of information on self-reported patient scores, and the absence of clinical equipoise in the opinion of both clinicians were the main limitations.

*Limitations and future work*: This paper is a systematic review that adheres to the PRISMA recommendations but excludes a more formal quantitative meta-analysis. This results from the observed heterogeneity of the identified studies as limitation can be considered the fact that only three online databases (PubMed, Scopus, and Semantic Scholar) were employed, and the exclusion of the grey literature may have led to the identification of a relatively small number of included studies.

Future works should use robust feature selection techniques and advanced AI (e.g., explainability models and graphical models) for knowledge extraction. This knowledge can be the input for powerful ML models. Furthermore, state-of-the-art DL models such as Siamese convolutional neural network (CNN) and transfer learning should be applied to biomechanical data from gait. 

AI is a valuable tool for identifying gait-related changes in post-knee surgery patients. The creation of robust explainable ML models for quantifying the recovery status during the rehabilitation process and the understanding of the contribution of the selected gait biomechanical parameters in the model’s output could lead to the creation of non-invasive and more powerful diagnostic and prognostic tools for clinicians. Hence, AI in the field of orthopedics may play a key role in forming new personalized rehabilitation interventions for the modification of abnormal gait patterns and subsequently avoid the development of knee OA.

## Figures and Tables

**Table 1:** A brief summary of the ML models presented in the papers in our survey.

Models	Description
**Logistic Regression (LR)**	LR is a supervised algorithm for predicting a binary outcome, such as yes or no, using prior observations of a dataset. Examining the relationship between one or more independent factors and a dependent variable, a logistic regression model predicts the dependent variable.
**Neural Network (NN)**	A neural network is a group of algorithms that attempts to identify underlying links in a set of data by mimicking the way the human brain works. The three basic components are the input layer, the processing layer, and the output layer. According to a variety of parameters, the inputs might be weighted. The processing layer has nodes and connections between these nodes that are supposed to be equivalent to neurons and synapses in an animal brain.
**XGBoost**	XGBoost belongs to the category of the gradient boosted tree algorithms. Gradient boosting predicts a target (dependent) variable by combining the estimates of several simpler, less accurate (weak) models.
**Random Forest (RF)**	RFs are used to tackle regression and classification problems in machine learning. It employs ensemble learning, that combines multiple classifiers in order to solve complex problems. RFs typically contain a number of different decision trees. The “forest” of the random forest can be trained via bagging or bootstrap aggregation. Bagging enhances machine learning accuracy with an ensemble meta-algorithm.
**Decision Trees (DTs)**	DTs belong to the supervised learning category and can be used for regression and classification tasks. The objective is to create a model capable of predicting the target variable accurately using a rule-based mechanism learned from the available data features. A tree can be viewed as an approximation that is piecewise constant.
**Naïve Bayes**	The Naïve Bayes algorithm is a category of supervised learning classifiers that are based on Bayes’ theorem. These classifiers use the “naïve” assumption that each pair of features is independent of one another regardless of the value of the class variable.
**Support vector machine (SVM)**	SVM aims to find such hyperplane that separates the data into two classes most effectively. There are numerous potential hyperplanes that might be selected to split the two data point samples. The main task of the SVM algorithm is to identify the hyperplane that maximizes the margin between the two classes, or the greatest distance between data points of different classes.
**K-nearest neighbors (KNN)**	The KNN algorithm is a supervised ML algorithm that may be applied to both regression and classification tasks. It works by (i) figuring out the distance between a testing sample and each data sample and (ii) picking the most common label as the predicted target in a classification task or combining the labels to obtain the output in a regression task.
**Hierarchical cluster analysis**	Hierarchical clustering, also known as hierarchical cluster analysis, is a technique that clusters related objects. The endpoint consists of a collection of clusters, where each cluster is distinct from the others and the items within each cluster are generally comparable. Objects are then combined in pairs based on the distance between them. The process continues with the next round of combining clusters. This process is repeated until there is only one cluster.

**Table 2:** Employed databases and search strategies.

Database	Search Strategy
**PubMed**	(“anterior cruciate ligament” OR “Total knee arthroplasty” OR “total knee replacement” OR Meniscectomy OR “Meniscal suturing” OR “unicompartment knee arthroplasty” OR “Anterolateral ligament reconstruction” OR “Anterolateral ligament repair” OR “Posterolateral corner reconstruction” OR “Posterior cruciate ligament” OR “High tibia osteotomy”) AND (walking OR gait) AND (“machine learning” OR “deep learning” OR “Artificial intelligence”) NOT images
**Scopus**	(“anterior cruciate ligament” OR “Total knee arthroplasty” OR “total knee replacement” OR “Meniscectomy” OR “Meniscal suturing” OR “unicompartment knee arthroplasty” OR “Anterolateral ligament reconstruction” OR “Anterolateral ligament repair” OR “Posterolateral corner reconstruction” OR “Posterior cruciate ligament” OR “High tibia osteotomy”) AND (walking OR gait) AND (“machine learning” OR “deep learning” OR “Artificial intelligence”) AND NOT (images)
**Semantic Scholar**	“anterior cruciate ligament” OR “Total knee arthroplasty” OR “total knee replacement” OR “Meniscectomy” OR “Meniscal suturing” OR “unicompartment knee arthroplasty” OR “Anterolateral ligament reconstruction” OR “Anterolateral ligament repair” OR “Posterolateral corner reconstruction” OR “Posterior cruciate ligament” OR “High tibia osteotomy” AND walking OR gait AND “machine learning” OR “deep learning” OR “Artificial intelligence” AND NOT images”

**Table 3:** Results of the employed studies.

Category	Author	Year	Task	Data	Subjects	Feature Engineering	Machine Learning	Validation	Results
TKA	Emmerzaal et al. [24]	2022	Classification	IMU	20 healthy controls, 19 with unilateral knee OA and 17 post TKA		LR	5-fold cross validation (CV)	67.3% Accuracy
	Young-Shand et al. [25]	2022	Classification	3D Gait analysis	135 pre TKA and 109 post TKA	Principal component analysis (PCA)	Hierarchical agglomerative (bottom-up) cluster analysis	-	-
	Jones et al. [26]	2016	Classification	3D Gait analysis	12 subjects with cruciate-retaining TKA, 12 subjects with mobile-bearing medial UKA and 121 healthy controls	-	DT	-	92% Accuracy at heathy group
	Martins et al. [27]	2015	Classification	IMU	TKA operated and control leg	Linear principal component analysis (PCA) and nonlinear kernel-PCA (KPCA)	Multiclass SVM	6-fold CV	98% Accuracy
	Kuntze et al. [28]	2015	Classification	EMG	10 unilateral gender-specific TKA and 9 healthy controls	Wavelet transform	SVM	leave-one-out cross-validation (LOOCV)	Biceps femoris (BF) recognition rate at 73.7% and vastus medialis (VM) recognition rate at 68.4%
ACL	Kokkotis et al. [29]	2022	Classification	3D Gait analysis	ACLD 44, ACLR 54 and healthy Control 53	ReliefF algorithm	XGBoost, RF, DT, Naïve Bayes, SVM, KNN, LR, NN	70% training and 30% testing	94.95% accuracy (SVM)

## Data Availability

Not applicable.
